# The impact of sarcopenia on sarcoma patients: a systematic review and meta-analysis

**DOI:** 10.1007/s11547-025-02016-9

**Published:** 2025-06-03

**Authors:** Domenico Albano, Moreno Zanardo, Mariachiara Basile, Nicole Alessandra De Micheli, Alessandro Berenghi, Francesca Serpi, Salvatore Gitto, Carmelo Messina, Luca Maria Sconfienza

**Affiliations:** 1 IRCCS Istituto Ortopedico Galeazzi, 20161 Milan, Italy; 2 Dipartimento di Scienze Biomediche, Chirurgiche ed Odontoiatriche, Università degli Studi di Milano, 20122 Milan, Italy; 3 Radiology Unit, IRCCS Policlinico San Donato, Via Morandi 30, 20097 San Donato Milanese, Italy; 4 Postgraduate School of Diagnostic and Interventional Radiology, Università degli Studi di Milano, Milan, Italy; 5 Dipartimento di Scienze Biomediche Per la Salute, Università degli Studi di Milano, 20122 Milan, Italy; 6 U.O.C. Radiodiagnostica, ASST Centro Specialistico Ortopedico Traumatologico Gaetano Pini-CTO, 20122 Milan, Italy

**Keywords:** Sarcoma, Systematic review, Meta-analysis, Mortality, Survival, Sarcopenia

## Abstract

**Purpose:**

Sarcopenia has been linked to poor outcomes in various cancers, but its specific effect on sarcoma patients remains unclear. This systematic review and meta-analysis investigates the impact of sarcopenia, estimated using CT, on sarcoma patients, focusing on prognostic implications and associated outcomes.

**Materials and methods:**

The PubMed, Embase, and SCOPUS databases were searched up to March 2025. Then, a meta-analysis of the data was performed. Overall survival (OS) and relapse-free survival (RFS) were the endpoints. Hazard ratios and 95% confidence intervals were assessed to evaluate the association between sarcopenia and survival of sarcoma patients.

**Results:**

Eighteen studies with a total of 1699 patients met the inclusion criteria. Liposarcoma was the most reported histotype in 67% of the studies, with extremities being the most common tumor location (50%), and chemotherapy was the primary intervention in 89% of cases, followed by radiation therapy (78%) and surgery (67%). Analyzing seven articles, a pooled HR of 1.91 (95% CI 1.09–3.34) for OS was reached, indicating that sarcopenic patients have a 91% higher risk of mortality compared to non-sarcopenic patients (*p* < 0.01). There is no evidence of selective publication (*p* = 0.137). The meta-analysis for the two studies that reported HR of RFS resulted 1.16 (95% CI 0.85–1.59), not significant (*p* = 0.28). The quality of the included studies demonstrated high methodological rigor.

**Conclusions:**

Worse outcomes have been observed in sarcopenic patients with sarcomas, but the impact of sarcopenia on OS and RFS still remains uncertain, highlighting the need for further research and standardized approaches.

*Trial Registration *The protocol for this review has been registered in the International Prospective Register of Systematic Reviews (PROSPERO) database (registration unique identifying number: CRD42024578969).

**Supplementary Information:**

The online version contains supplementary material available at 10.1007/s11547-025-02016-9.

## Introduction

Sarcopenia, as defined by the European Working Group on Sarcopenia in Older People, is a progressive skeletal muscle disorder that heightens the risk of adverse events [1]. While it was once considered just an inevitable consequence of aging, current understanding recognizes that multiple factors contribute to its onset [2]. Additionally, sarcopenia can develop secondarily to systemic diseases, particularly those associated with inflammatory processes like cancer or organ failure. In various surgical and medical disciplines, sarcopenia has been identified as a predictor of poor outcomes [3–7]. Sarcopenic patients, for instance, show reduced efficacy in responding to chemotherapy, leading to increased frailty, treatment toxicity, diminished quality of life, and heightened postoperative morbidity and mortality [8–10].

Muscle mass or quantity can be estimated through various techniques. Dual-energy X-ray absorptiometry is considered the most widely used method [11], but CT and MRI are regarded as optimal techniques for opportunistic retrospective assessment [12–14]. In particular, CT is favored for cancer patients due to its widespread availability and frequent use in assessing tumor staging and follow-up restaging [15]. Numerous studies and extensive meta-analyses have examined the role of muscle mass, as identified on CT, in predicting cancer patient outcomes at diagnosis, during perioperative care, and throughout survivorship [16, 17]. Nevertheless, evidence-based literature specifically addressing the role of sarcopenia in sarcomas, which are histologically distinct, remains comparatively scarce [9].

Sarcomas encompass a group of tumors, characterized by various histological subtypes originating from mesenchymal or connective tissue. Soft tissue sarcomas represent about 0.8% of all cancers in the USA and are among the top five causes of cancer-related deaths in individuals under 20; annually, sarcomas account for an estimated 13,000 to 16,000 new cases and 5,000 to 6,000 deaths in the USA [18]. Today, treatment for sarcomas mostly involves surgical resection, which remains the cornerstone of curative treatment, but chemotherapy and radiation therapy are often included in preoperative, intraoperative, or postoperative settings. Therefore, identifying biomarkers, such as muscle mass assessed via CT, that could predict survival rates, tumor recurrence, and post-surgical complications, would be beneficial for managing these patients [19]. Recent studies have begun to explore the relationship between sarcopenia and both survival rates and postoperative complications in sarcoma patients [20, 21].

This systematic review and meta-analysis aim to investigate the current evidence on sarcopenia in sarcoma patients, highlighting its prevalence, clinical and prognostic implications, as well as the associated complications and outcomes.

## Materials and methods

### Study design

This systematic review was conducted in accordance with the Preferred Reporting Items for Systematic Reviews and Meta-Analyses (PRISMA) [22]. The protocol for this review has been registered in the International Prospective Register of Systematic Reviews (PROSPERO) database (registration unique identifying number: CRD42024578969).

### Literature search strategy

We performed a comprehensive literature search in the PubMed, Embase, and SCOPUS databases from their inception until March 2025. The search terms used included “sarcopenia” or “muscle mass” and “sarcoma” and their expansions, and the search was limited to English-language publications. Studies were initially screened by title and abstract, followed by a full-text review of eligible studies. We also thoroughly reviewed the references of the included studies and relevant reviews to identify any additional eligible publications. The search was conducted by two independent reviewers with three years of experience in systematic reviews and was supervised and revised by a senior researcher with 10 years of experience.

### Inclusion and exclusion criteria

The inclusion criteria for this review were as follows: (i) studies involving human participants; (ii) studies reporting outcome data on survivorship or postoperative complications, with results expressed in hazard ratio (HR) with 95% confidence intervals (CI) comparing sarcopenic and non-sarcopenic patients with sarcoma. Exclusion criteria included: (i) case reports, case series with fewer than ten subjects, narrative reviews, guidelines, consensus statements, editorials, letters, comments, or conference abstracts; (ii) studies with insufficient outcome data.

### Data extraction

Data extraction was performed independently by two reviewers (M. B. and N. D. M.) using a standardized extraction form through Microsoft Excel. Data extracted included author information, year of publication, study design (prospective or retrospective), sample size (number of subjects and sarcopenic patients), characteristics of the study population (such as age, gender, and sarcoma type), interventions (e.g., surgery, chemotherapy, radiation therapy), and outcomes of interest—including overall survival (OS) and relapse-free survival (RFS).

For studies that reported HRs with corresponding 95% confidence intervals (CIs), these data were extracted directly. Where necessary, data were converted or calculated from the available information to ensure consistency across studies. Any discrepancies between reviewers were resolved through discussion, with a third reviewer consulted if needed (D. A.). All extracted data were double-checked for accuracy before being included in the final analysis.

### Quality assessment

The quality of included studies was assessed independently by two researchers, with any disagreements resolved through discussion. The QualSyst Tool for the Assessment of Quality of Included Studies [23] was employed to evaluate the quality of the observational studies. This tool contains 10 items, with study quality scored on a scale from 0 to 2. A score of 0 indicates that the item was not described in the paper, 1 indicates inadequate description, and 2 indicates an adequate description.

### Statistical analysis

The primary outcomes of interest included OS and RFS in sarcopenic versus non-sarcopenic patients with sarcoma. DerSimonian and Laird random effects meta-analysis [24] to pool HRs along with the corresponding 95% CIs was used due to the anticipated substantial heterogeneity considering the possible variation in types, localization and stages of sarcoma and cut‐off values for the definition of sarcopenia. Heterogeneity among the studies was assessed using the *I*^2^ statistic and Cochran’s *Q* test, with an *I*^2^ value greater than 50% indicating substantial heterogeneity [25]. Funnel plot and Egger’s test were used to detect publication bias in studies reporting overall survival [26]. Meta-analysis was performed using MetaAnalysisOnline.com tool [27]. Statistical significance was set at *p* < 0.05 for all analyses [28].

## Results

**Fig. 1:**
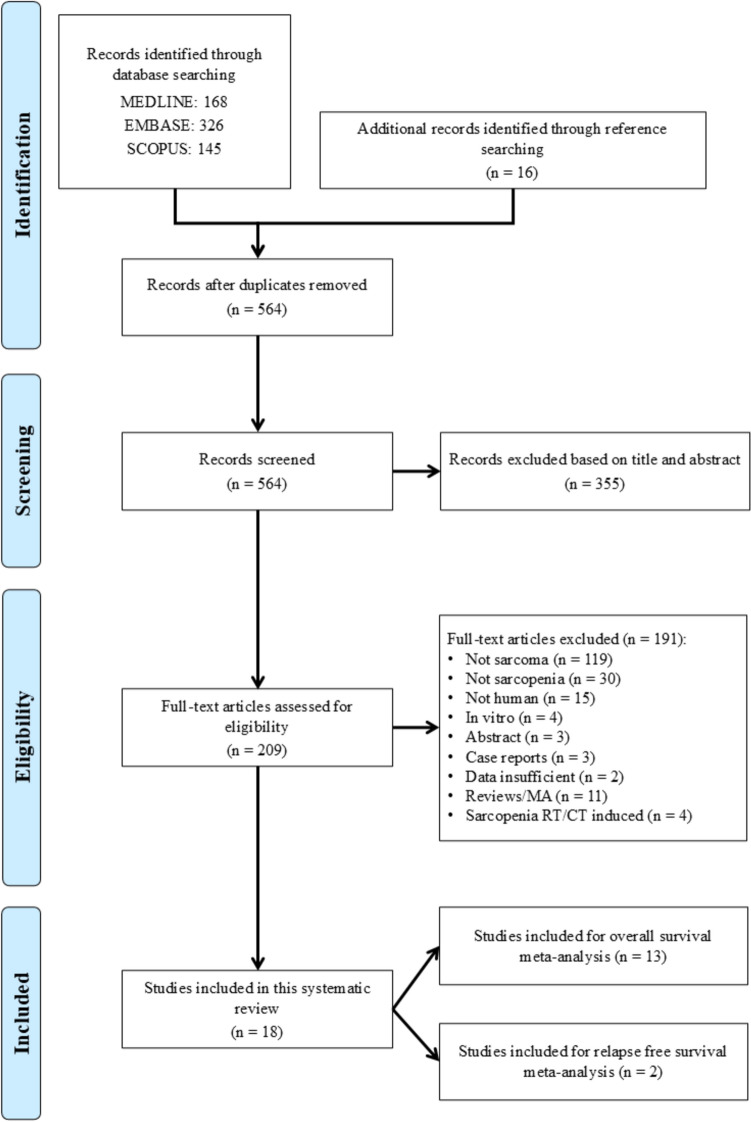
Flowchart of study selection

A search of the databases yielded a total of 356 studies. After the removal of duplicates and initial screening of titles and abstracts, 110 full-text articles were assessed for eligibility. Of these, 18 studies [9, 20, 21, 29–43] met the inclusion criteria. The study selection process is summarized in the PRISMA flow diagram (Fig. 1). These studies spanning from 2015 [20] to 2024 [41] investigated the impact of sarcopenia on OS in patients with various types of sarcoma, with a total sample size of 1,699 patients, ranging from 21 [39] to 163 [31] subjects. The included studies were primarily retrospective in design, with the exception of one study that was conducted prospectively [40].

The studies covered a diverse range of sarcoma types, including liposarcoma reported in 12/18 (67%) articles, leiomyosarcoma in 11/18 (61%), synovial sarcoma in 7/18 (39%), pleomorphic sarcoma in 10/18 (56%), Ewing sarcoma in 3/18 (17%), and others in 12/18 (67%). Sarcoma localization was generally categorized into extremities, trunk, and other regions. Extremities were the most common localization for sarcomas in the included studies (9/18, 50%), reflecting the typical distribution of soft tissue sarcomas, while 8/18 (50%) articles in trunk or 5/18 (28%) articles in other regions. Patients with metastasis were reported in 4/18 articles (22%), highlighting the presence of advanced disease in the studied populations.

Surgery was reported in 12/18 (67%) articles. Surgery alone was the focus in a few studies, while others combined it with additional therapies. Radiation therapy was mentioned in 14/18 (78%) articles. This modality was often used in combination with surgery, particularly in cases where local control of the tumor was crucial or when the sarcoma was in regions where complete surgical resection was challenging. Also, chemotherapy was reported in 16/18 (89%) articles, typically administered in cases of high-grade sarcomas, metastatic disease, or as an adjunct to surgery. All data are reported in Tables 1, 2, and 3.

**Table 1:** Article characteristics

Author year	Year	DOI	Study design	Subjects (n)	Sarcopenic subjects (n)	Female (%)
Ramanovic 2024	2024	10.2478/raon-2024-0013	Retrospective	58	19	41
Buğdaycı 2023	2023	10.1007/s00247-022-05583-5	Retrospective	60	34	48.4
Nasirishargh 2023	2023	10.1002/jso.27199	Prospective	65	14	51
Ban 2022	2022	10.21873/cdp.10094	Retrospective	76	41	
Boyle 2022	2022	10.1016/j.amjsurg.2021.08.005	Retrospective	95		44
Casirati 2022	2022	10.1016/j.clnesp.2022.02.125	Retrospective	100	60	52
Romano 2022	2022	10.3390/nu14020383	Retrospective	21	12	47.6
Telli 2022	2022	10.1080/01635581.2022.2063349	Retrospective	79	40	47
Zhao 2022	2022	10.1186/s12957-022-02846-1	Retrospective	72	42	35
Brinkmann 2021	2021	10.1002/jso.26776	Retrospective	31	13	
Jo 2021	2021	10.21873/anticanres.14959	Retrospective	147		47
Phan 2021	2021	10.1002/jso.26077	Retrospective	89		40
Strassmann 2021	2021	10.1007/s10147-021-01997-7	Retrospective	89	28	55
Boutin 2020	2020	10.21037/qims.2020.02.09	Retrospective	137		45
Hendrickson 2020	2020	10.1002/jso.25898	Retrospective	145	38	43
Hirai 2020	2020	10.1093/jjco/hyaa100	Retrospective	163		40.5
Veld 2016	2016	10.1007/s00330-016-4306-6	Retrospective	135		36
Wilson 2015	2015	10.1155/2015/146481	Retrospective	137		50

**Table 2:** Sarcoma characteristics

Author year	Grade					Type of sarcoma						MTS (n)
	1 (n)	2 (n)	3 (n)	4 (n)	Unknown (n)	Liposarcoma (n)	Leiomyosarcoma (n)	Synovial sarcoma (n)	Pleomorphic sarcoma (n)	Ewing sarcoma (n)	Other (n)	
Ramanovic 2024	9	30	16	3		35	9		1		13	
Buğdaycı 2023										34	26	12
Nasirishargh 2023	27	6	32			44	10				11	89
Ban 2022						8			17		51	
Boyle 2022	57	19	8		11	34	16		10		35	
Casirati 2022						71	13					
Romano 2022										14	7	9
Telli 2022	6	19	54			14	6	9	18		32	
Zhao 2022	31	27	14			72			1			
Brinkmann 2021												
Jo 2021	10	10	115		12		18	15	27	19	68	
Phan 2021		9	80									
Strassmann 2021	5		74			10	7	27			55	
Boutin 2020	51		86			43	11	9	18		56	
Hendrickson 2020	16	19	110			11	12	7	32		83	14
Hirai 2020	8		155			34	8	9	60		52	
Veld 2016												
Wilson 2015		15	97			30	16	7	57			

*MTS* metastasis

**Table 3:** Disease stage, sarcoma localization, and therapy

Author year	Stage					Localization			Therapy		
	1 (n)	2 (n)	3 (n)	4 (n)	Unknown (n)	Extremity	Trunk	Other	Surgery (%)	Radiation therapy (%)	Chemotherapy (%)
Ramanovic 2024	24		34			74	15		100	59.5	26
Buğdaycı 2023						38	3	19			100
Nasirishargh 2023										58.4	100
Ban 2022	5	22	40	8	1	54	22		91		20
Boyle 2022	16	21	18	5			46	49		32.6	29.4
Casirati 2022									100	25	24
Romano 2022											
Telli 2022	6	11	59		3				100	51	49
Zhao 2022											
Brinkmann 2021									100	47	35
Jo 2021	6	24	47	44	3	101	43	3	84	51	71
Phan 2021		20	60	9		74	15		100	59.5	24
Strassmann 2021									58.4	58.4	100
Boutin 2020	47	23	55	12		67	21	49	87	25	9
Hendrickson 2020						145				19	32
Hirai 2020	8	33	118	4		135	8	20	100	34.4	5
Veld 2016	19	63	29	21		135			66	49	12.5
Wilson 2015	34	27	33	42					100	61	21

### Meta-analysis

**Fig. 2:**
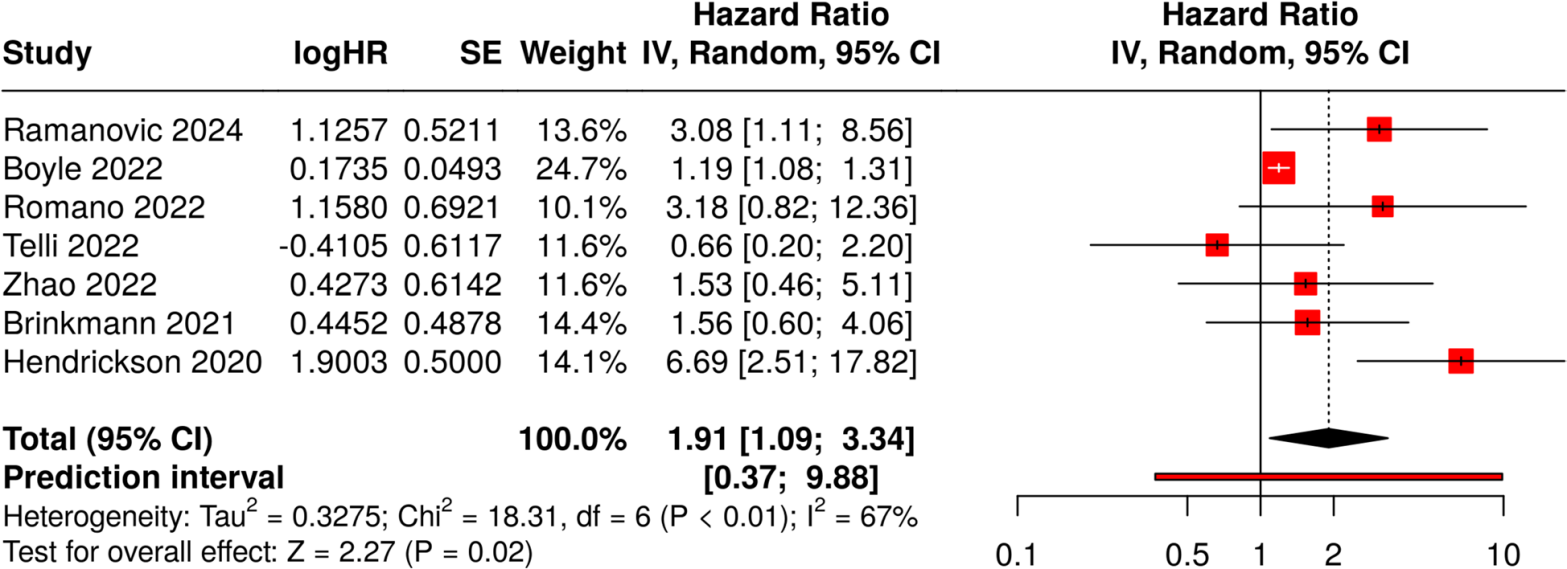
Forest plot representing the individual hazard ratios for overall survival for each study and the pooled estimate. The plot also includes the confidence intervals for each study and the overall pooled HR

The meta-analysis of the seven studies reporting HR for OS (Table 4) resulted in a pooled HR of 1.91 (95% confidence interval 1.09–3.34), indicating that sarcopenic patients have a 91% higher risk of mortality compared to non-sarcopenic patients (*p* < 0.001), as shown in the forest plot reported in Fig. 2. A significant heterogeneity was present (*p* = 0.001), indicating variable effects in scale and/or direction. An I^2^ value of shows that 67% of the differences among the cohorts arises from heterogeneity rather than arbitrary chance.

**Table 4:** Hazard ratios and 95% confidence intervals for overall survival and relapse-free survival

Author year	Subjects (n)	Sarcopenic subjects (n)	HR (OS)	95% CI Low	95% CI High	HR (RFS)	95% CI Low	95% CI High
Ramanovic 2024	58	19	3.18	1.11	8.56			
Buğdaycı 2023	60	34						
Nasirishargh 2023	65	14						
Ban 2022	76	41						
Boyle 2022	95		1.19	1.08	1.31			
Casirati 2022	100	60						
Romano 2022	21	12	3.19	0.82	12.36			
Telli 2022	79	40	0.80	0.2	2.2			
Zhao 2022	72	42	1.54	0.463	5.106			
Brinkmann 2021	31	13	1.57	0.6	4.06			
Jo 2021	147		0.99	0.99	0.99	0.99	0.99	0.99
Phan 2021	89		0.61	0.43	0.86			
Strassmann 2021	89	28				2.04	0.68	6.12
Boutin 2020	137		0.87	0.81	0.87			
Hendrickson 2020	145	38	6.68	2.506	17.823	1.11	1.03	1.2
Hirai 2020	163					0.68	0.49	0.95
Veld 2016	135		0.96	0.94	0.99			
Wilson 2015	137							

HR, hazard ratio; OS, overall survival; CI, confidence interval; RFS, relapse-free survival

**Fig. 3:**
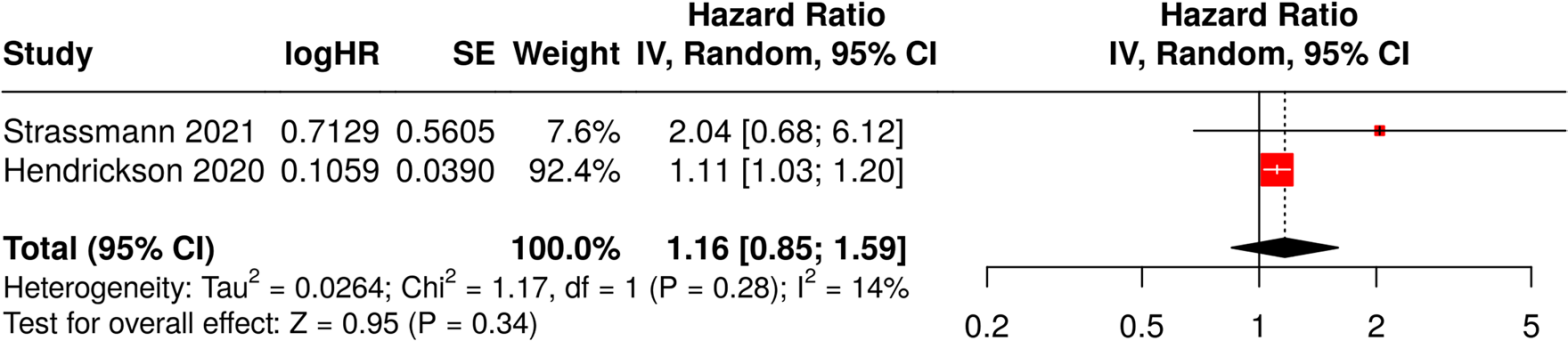
Forest plot representing the individual hazard ratios for relapse-free survival for each study and the pooled estimate. The plot also includes the confidence intervals for each study and the overall pooled HR

The meta-analysis of the two studies reporting HR for RFS resulted 1.16 (0.85 to 1.59). This indicates a very high degree of uncertainty in the pooled estimate, and the test for overall effect does not show a significant effect (*p* = 0.340). We did not observe significant heterogeneity (*I*^2^ = 14%), suggesting that the effect sizes across studies were consistent in both magnitude and direction. Available data are not sufficient to provide a precise estimate, as reported in the forest plot Fig. 3 and Table 4.

### Publication bias

**Fig. 4:**
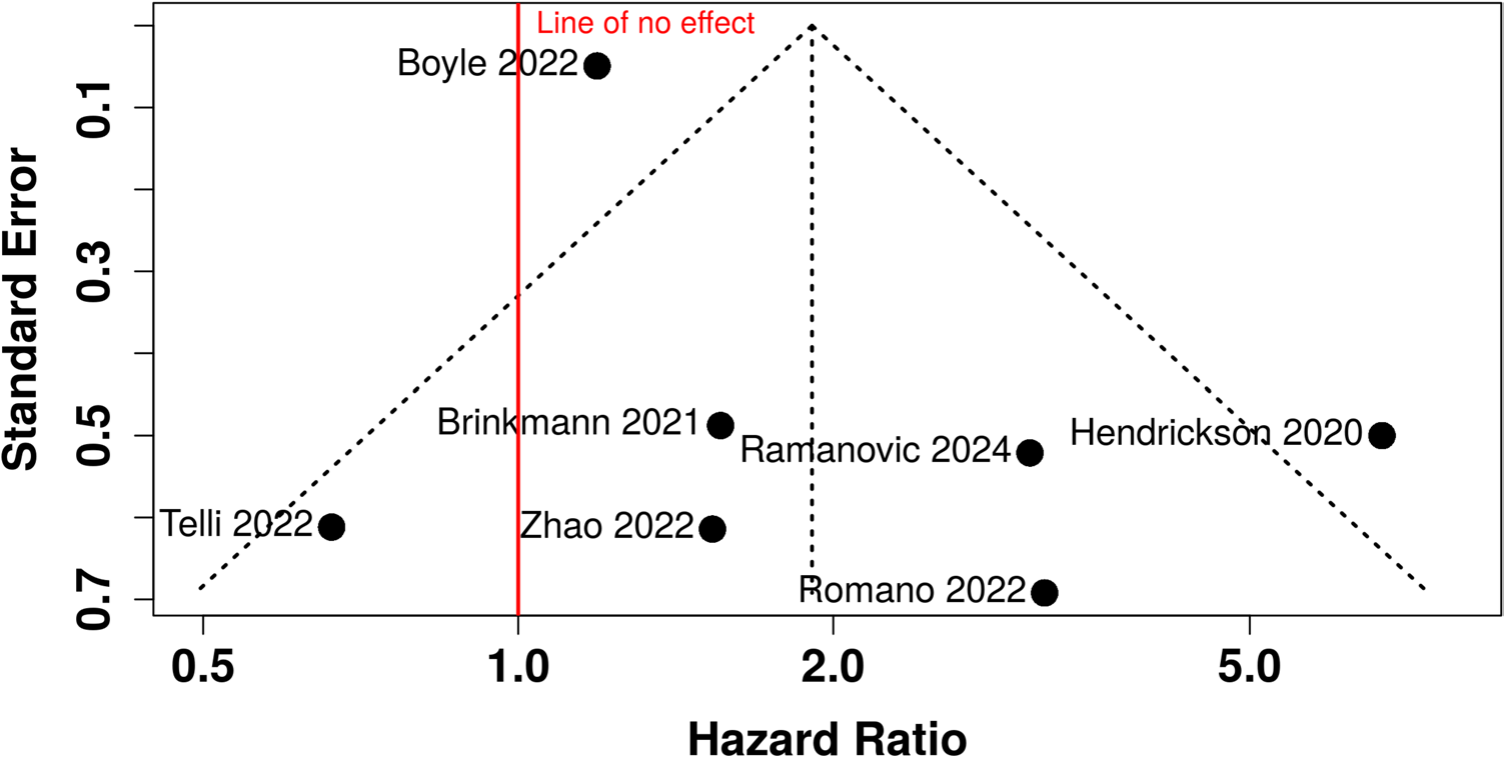
Funnel plot assessing publication bias in the meta-analysis of sarcopenia’s impact on sarcoma outcomes. The funnel plot does not indicate a potential publication bias. The Egger’s test does not support the presence of funnel plot asymmetry (*p* = 0.137) for the OS analysis. The vertical red line represents no effect, and the spread of points indicates variability in the standard errors of the reported hazard ratios

The funnel plot analysis (Fig. 4) does not indicate a potential publication bias. The Egger’s test does not support the presence of funnel plot asymmetry (*p* = 0.137) for the OS analysis. These data suggests that studies examining the association between sarcopenia and outcomes in sarcoma patients are not selectively published based on the significance of their findings. Both significant and non-significant results appear to be equally represented in literature.

### Quality assessment of included studies

The studies demonstrated high methodological rigor, with scores ranging from 15 to 20 out of 20. Studies by Ramanovic et al. [41] Brinkmann et al. [34], and Phan et al. [33] achieved perfect scores, indicating strong adherence to scientific standards. However, a few studies, such as Wilson et al. [20] and Veld et al. [29], scored lower due to less detailed descriptions of their data collection methods and sampling strategies.

## Discussion

This systematic review and meta-analysis aimed to investigate the impact of sarcopenia on OS and RFS in patients with sarcoma. The results highlight several challenges in drawing definitive conclusions about the prognostic significance of sarcopenia in this patient population.

The relationship between sarcopenia and outcomes in sarcoma patients reveals a complex and heterogeneous picture. Preoperative sarcopenia and hypoalbuminemia are associated with worse OS in certain subgroups, such as patients with retroperitoneal sarcoma [40, 41]. However, the prognostic value of sarcopenia is not universally consistent across all sarcoma types and patient populations. For instance, in children with Ewing sarcoma or osteosarcoma, the association between sarcopenia and survival remains inconclusive, highlighting the need for further research in pediatric populations [21, 39]. In adult sarcoma patients, sarcopenia has been linked to worse surgical outcomes, including increased wound complications, although its impact on OS and RFS is less definitive [38]. Muscle area and attenuation metrics are associated with OS in some studies, particularly in patients with soft tissue sarcoma and advanced/metastatic disease [32]. It can be postulated that the impact of sarcopenia might be more pronounced in advanced disease stages, in which higher levels of inflammation can be associated with cancer cachexia [44]. Nevertheless, certain studies indicate that sarcopenia, as assessed by specific skeletal muscle measurements, does not predict survival or postoperative complications in localized soft tissue sarcoma [45]. The pooled HR for OS from the included studies indicated that sarcopenic patients have a significant 91% higher risk of mortality compared to their non-sarcopenic counterparts (HR 1.91; 95% confidence interval 1.09–3.34). This trend was consistent across various studies, highlighting that sarcopenia may be considered as a potential prognostic factor. Studies such as Buğdaycı et al. [21] and Hendrickson et al. [30] reported similar trends but lacked sufficient data to support statistical significance, indicating that the observed increased risk might be present but remains uncertain.

**Fig. 5:**
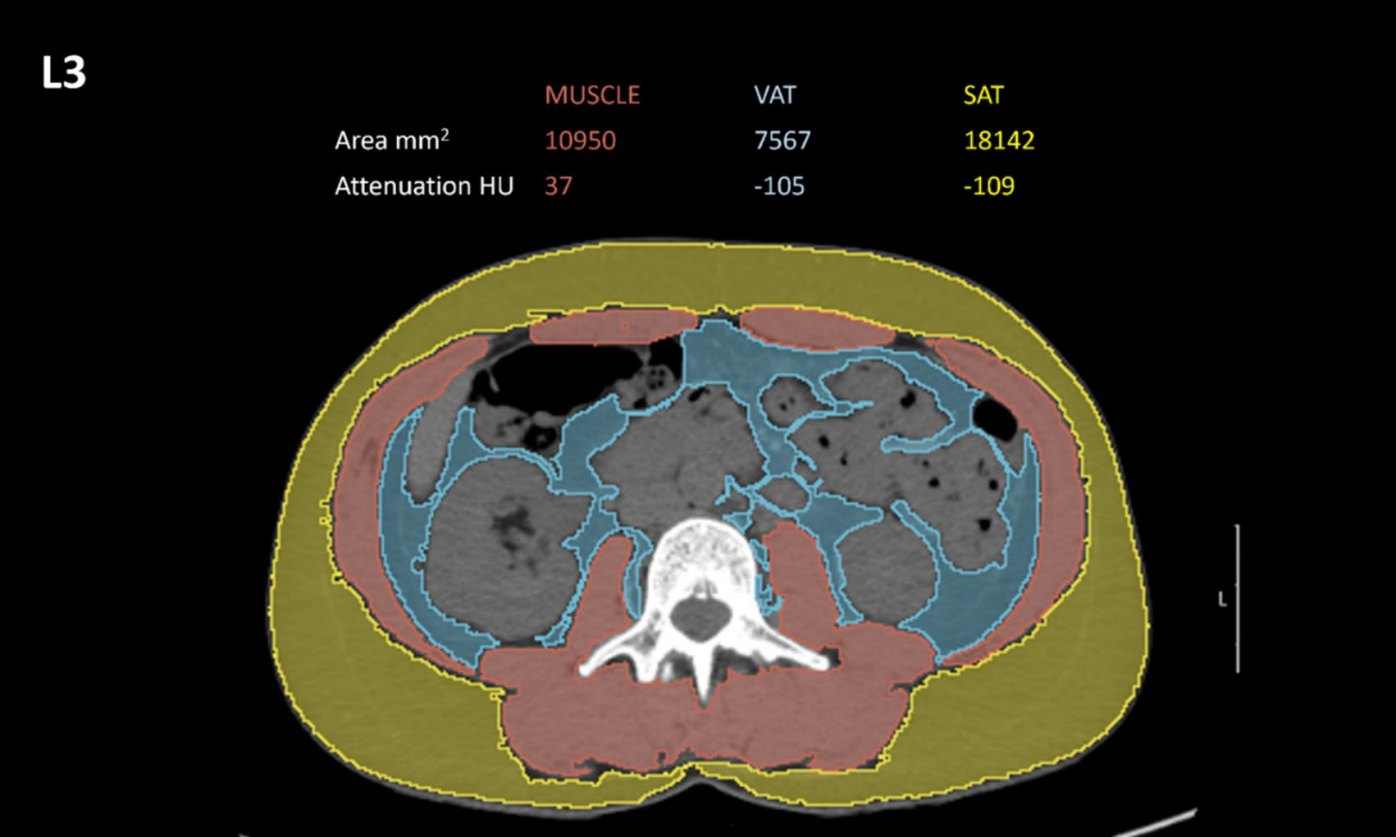
Axial CT images obtained at L3 level in 49-year-old woman which was used to determine skeletal muscle area (SMA). SMA measured 109.5 cm^2^. Blue indicates automated segmentation of visceral adipose tissue (VAT), and yellow denotes automated segmentation of subcutaneous adipose tissue (SAT)

**Fig. 6:**
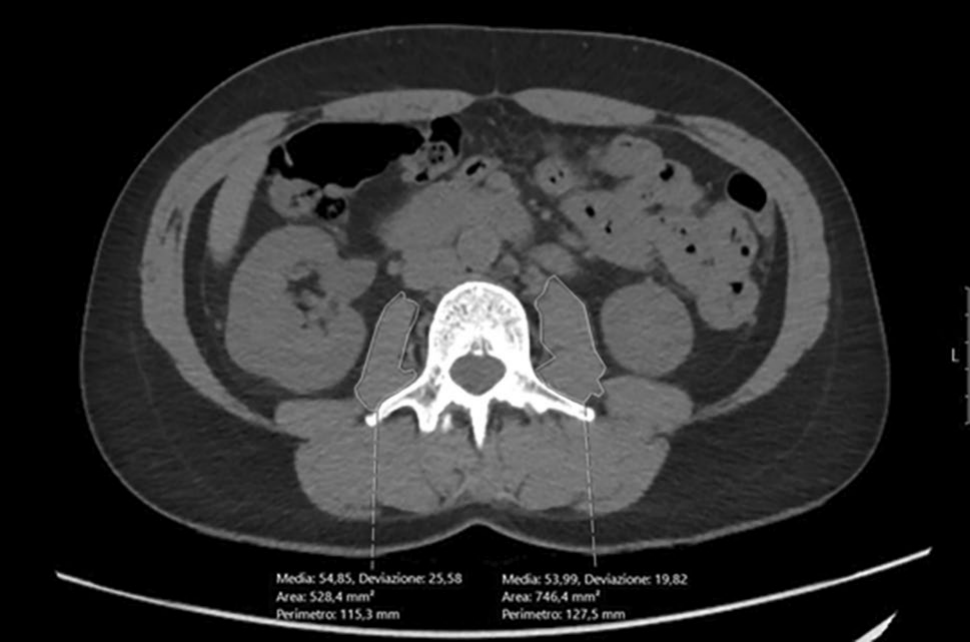
Computerized tomography (CT) abdomen image of a 49-year-old patient showing standardized measurements of the psoas area (mm2) and density (HU)

Several CT-derived imaging biomarkers can be used for the evaluation of muscle quantity and quality, obtained by drawing a region of interest (ROI) on CT slices to measure cross-sectional area and/or attenuation values. Measurements can involve different muscle groups at various levels, although most segmentations have been done at the L3 or L4 level (Fig. 5) and ROIs include generally psoas muscles (Fig. 6) [46]. The high heterogeneity in measurement methods among the examined studies as well as the lack of standardization of threshold values for determining sarcopenia probably help explaining the lack of statistical significance. For instance, sarcopenia was determined by Ban et al. [42] as a total psoas area/m^2^ ≤ 5.0 cm^2^/m^2^, while in the study by Hendrickson et al. [29], sarcopenia was defined as psoas index < 5.45 cm^2^/m^2^ for men and < 3.85 cm^2^/m^2^ for women. Furthermore, most studies did not provide a threshold value. Notably, the muscles to be investigated may vary according to the location of sarcomas, any surgical interventions (since implants may generate metal-derived artifacts), and availability of unenhanced images, since muscle density is affected by the enhancement after contrast agent administration [47]. Cross-sectional area is often indexed by height to use skeletal muscle index as a more accurate imaging parameter of sarcopenia. In this setting, a systematic review reported values of 52–55 cm^2^/m^2^ in men and 39–41 cm^2^/m^2^ in women as the most common skeletal muscle index threshold values to be applied on CT for the evaluation of sarcopenia [48].

Some reasons may explain differences related to age. First, the relatively low number of pediatric patients with sarcoma included in studies on sarcopenia. Then, skeletal muscle index can be a less powerful imaging biomarker in children than in adults, given that skeletal muscle might scale differently with respect to height in pediatric series. In pediatric populations, such as those reported by Buğdaycı et al. [21], the impact of sarcopenia was less evident or inconsistent, considering also that pediatric patients have overall better tolerance to surgery that might mask the effects of sarcopenia. Further, another confounding factor might be the increasing fat mass in girls and muscle mass in males during puberty [49]. Moreover, the studies included a diverse range of sarcoma subtypes, including liposarcoma, leiomyosarcoma, and Ewing sarcoma, with varying anatomical locations (extremities, trunk, and others). This diversity contributed to substantial heterogeneity (*I*^2^ = 67%), making the aggregation of findings challenging. For example, studies focusing on extremity sarcomas, like those by Boyle [35], often observed higher rates of postoperative complications among sarcopenic patients, particularly those undergoing surgery combined with chemotherapy. In contrast, outcomes for sarcomas located in the trunk or other regions varied more widely [50].

The influence of sarcopenia may vary depending on tumor location and the type of sarcoma [9]. For example, central sarcopenia has been associated with local tumor recurrence in patients undergoing sacral tumor resection [34], while low skeletal muscle gauge did not significantly impact survival outcomes, but was linked to a higher incidence of surgical wound complications in other studies [20, 31, 34, 42]. Hendrickson et al. [30] specifically highlighted that sarcopenia was associated with increased mortality, but not necessarily with postoperative complications in extremity sarcomas, suggesting that the impact of sarcopenia might vary depending on tumor location and surgical approach. It should be highlighted that concerning chemotherapy, lower dosage and thereby less aggressive treatment are administered to sarcopenic patients than non-sarcopenic [51]. Hence, a preference for less intensive treatment in sarcopenic patients is already being made based on clinical judgment. This can partly justify the worse response to treatment observed in sarcopenic patients [9].

In metastatic settings, sarcopenia was a stronger predictor of poor outcomes. Nasirishargh et al. [40] demonstrated that sarcopenic patients with metastatic sarcomas had significantly reduced survival, likely due to the compounded effects of tumor burden, systemic inflammation, and reduced physical resilience [52]. Several studies, including Zhao [38], identified sarcopenia as a predictor of worse surgical outcomes [53], such as increased wound complications and longer hospital stays [54]. This impact was particularly evident in high-grade or metastatic sarcomas, where treatment complexity and tumor burden were higher. RFS analysis was limited by the number of studies that reported HRs. The pooled HR for RFS suggested a trend toward worse outcomes for sarcopenic patients, but the results were highly uncertain due to substantial variability in measurement techniques and patient follow-up durations. Hence, sarcopenia might be a worse prognostic factor indicating an increased risk of mortality in sarcoma patients, probably related to the established idea concept that sarcopenic patients have reduced reserves, decreasing survival and making them more susceptible to postoperative complications, whether due to disease or frailty. Nevertheless, it still seems to be a weak risk stratification tool to predict the risk of post-surgical complications and mortality to be used for decision making. Furthermore, we are aware that skeletal muscle status can be reliably assessed through several imaging modalities, but literature on effective interventions to decrease the risk associated with sarcopenia in sarcoma patients is still scarce. Suggested intervention strategies involve optimizing nutrition and incorporating physical therapy in the preoperative or perioperative phases for elective surgeries. While these approaches may be challenging for patients requiring immediate surgery, several sarcoma patients undergo therapies for a long time, offering an opportunity for intervention. Studies have reported interesting preliminary data on branched-chain amino acid supplementation for decreasing sarcopenia and infectious complications after orthopedic surgical interventions [55, 56]. Nevertheless, these findings should not be directly applied to sarcoma patients, so prospective trials are needed in this specific population. Notably, considering conflicting data on the effects of calorie restriction, fasting, and dedicated low-carb high-fat diets on tumorigenesis, it is crucial to balance possible short-term advantages against long-term risks of tumor relapse [57].

Some limitations must be pointed out. The moderate high *I*^2^ value (67%) indicates a moderate to high level of variability among the included studies, suggesting that differences in study design, populations, or methods may be influencing the overall results. This level of heterogeneity suggests that the pooled estimate should be interpreted with caution, as the differences between studies could be due to varying study designs, populations, measurement methods, or other factors. In particular, the included studies involve different patient populations with varying characteristics (e.g., age, sarcoma subtypes, stage of disease), which can introduce variability in the results. The overall sample size and the number of relapse events reported in the included studies were relatively small, increasing the likelihood of random error influencing the results. Another limitation was the variability of how sarcopenia and RFS were measured across the studies. Some studies utilized CT-based assessments of muscle mass, while others relied on different diagnostic criteria, contributing to variability in reported outcomes. Differences in imaging modalities, cutoff values for muscle mass, and follow-up periods can all affect the comparability of results. Additionally, variations in how RFS is defined and measured—such as differences in the timing of follow-up or criteria for defining relapse—can introduce further inconsistencies. Furthermore, new techniques for evaluating sarcopenia, such as 3D segmentation of subcutaneous and abdominal fat and psoas muscles, suggest that research is progressing in this area [58, 59]. Recent surveys, such as the 2024 EuroAIM/EuSoMII study, highlight growing interest in applying artificial intelligence tools to improve body composition analysis, particularly for automated muscle segmentation and standardized extraction of sarcopenia-related imaging biomarkers [60]. Nevertheless, it should be noted that most of the studies provided credible and well-supported conclusions, ensuring the reliability of the meta-analysis outcomes.

In conclusion, worse outcomes have been observed in sarcopenic patients with sarcomas. The impact of sarcopenia on OS and RFS still remains uncertain, particularly in pediatric populations, although better outcomes seem to be reported in non-sarcopenic adult patients, especially in those with soft tissue sarcoma and advanced/metastatic disease. Given the heterogeneity of sarcomas and the complex interactions between sarcopenia and cancer biology, further research is necessary to establish standardized diagnostic/prognostic criteria and intervention strategies. It is essential to conduct further research with larger, more homogenous patient cohorts and standardized measurement techniques. Future studies should aim to standardize the definition and assessment of sarcopenia and consider the specific biological characteristics of different sarcoma subtypes.

## Supplementary Information

Below is the link to the electronic supplementary material.

Supplementary file 1 (DOCX 20 kb)

## Data Availability

The datasets analyzed during the current study are available from the corresponding author upon reasonable request. Access to data will be provided in compliance with applicable ethical guidelines and institutional regulations.

## Abbreviations

CI: Confidence interval
HR: Hazard ratio
OS: Overall survival
PRISMA: Preferred reporting items for systematic reviews and meta-analyses
RFS: Relapse-free survival
ROI: Region of interest
SMI: Skeletal muscle index
