# Supplementary material for: The impact of sarcopenia on sarcoma patients: a systematic review and meta-analysis
Source: Radiol Med. 2025 Jun 3;130(9):1373–85. doi: 10.1007/s11547-025-02016-9 (PMC12454457; doi:10.1007/s11547-025-02016-9)
Supplement: Supplementary file 1 — (DOCX 20 kb) [file 11547_2025_2016_MOESM1_ESM.docx]

| Criteria | Question/objective sufficiently described? | Study design evident and appropriate? | Context for the study clear? | Connection to a theoretical framework/wider body of knowledge? | Sampling strategy described, relevant, and justified? | Data collection methods clearly described and systematic? | Data analysis clearly described and systematic? | Use of verification procedure(s) to establish credibility? | Conclusions supported by the results? | Reflexivity of the account? | Total |
| --- | --- | --- | --- | --- | --- | --- | --- | --- | --- | --- | --- |
| Ramanovic 2024 | 2 | 2 | 2 | 2 | 2 | 2 | 2 | 2 | 2 | 2 | 20 |
| Buğdaycı 2023 | 2 | 2 | 2 | 1 | 2 | 1 | 1 | 1 | 2 | 2 | 16 |
| Nasirishargh 2023 | 2 | 2 | 2 | 2 | 2 | 2 | 2 | 1 | 2 | 2 | 19 |
| Ban 2022 | 2 | 1 | 2 | 2 | 1 | 2 | 1 | 1 | 2 | 2 | 16 |
| Boyle 2022 | 2 | 2 | 2 | 2 | 2 | 2 | 2 | 1 | 2 | 2 | 19 |
| Casirati 2022 | 2 | 2 | 2 | 2 | 2 | 2 | 1 | 1 | 2 | 2 | 18 |
| Telli 2022 | 2 | 2 | 2 | 2 | 2 | 2 | 2 | 1 | 2 | 2 | 19 |
| Romano 2022 | 2 | 2 | 2 | 2 | 2 | 2 | 2 | 1 | 2 | 2 | 17 |
| Zhao 2022 | 2 | 2 | 2 | 2 | 2 | 2 | 2 | 1 | 2 | 2 | 19 |
| Brinkmann 2021 | 2 | 2 | 2 | 2 | 2 | 2 | 2 | 2 | 2 | 2 | 20 |
| Jo 2021 | 2 | 2 | 1 | 2 | 2 | 2 | 2 | 1 | 2 | 1 | 17 |
| Phan 2021 | 2 | 2 | 2 | 2 | 2 | 2 | 2 | 2 | 2 | 2 | 20 |
| Strassmann 2021 | 2 | 2 | 1 | 2 | 2 | 2 | 2 | 1 | 2 | 2 | 18 |
| Boutin 2020 | 2 | 2 | 2 | 2 | 2 | 2 | 1 | 1 | 2 | 2 | 18 |
| Hendrickson 2020 | 2 | 2 | 2 | 2 | 2 | 1 | 1 | 1 | 2 | 2 | 17 |
| Hirai 2020 | 2 | 2 | 2 | 2 | 1 | 2 | 2 | 1 | 2 | 2 | 19 |
| Veld 2016 | 2 | 2 | 2 | 2 | 1 | 1 | 1 | 1 | 2 | 2 | 16 |
| Wilson 2015 | 2 | 2 | 2 | 1 | 1 | 1 | 1 | 1 | 2 | 2 | 15 |
